# Limited sex-biased neural gene expression patterns across strains in Zebrafish (*Danio rerio*)

**DOI:** 10.1186/1471-2164-15-905

**Published:** 2014-10-17

**Authors:** Ryan Y Wong, Melissa M McLeod, John Godwin

**Affiliations:** Department of Biological Sciences, W.M. Keck Center for Behavioral Biology, North Carolina State University, Box 7617, Raleigh, NC 27695-7617 USA; Department of Biology, University of Nebraska at Omaha, Omaha, NE 68182 USA

**Keywords:** Sexual dimorphism, Sexual plasticity, Brain, *Danio rerio*, Sex, Gene expression, Transcriptome, RNA-sequencing, Gene coexpression network

## Abstract

**Background:**

Male and female vertebrates typically differ in a range of characteristics, from morphology to physiology to behavior, which are influenced by factors such as the social environment and the internal hormonal and genetic milieu. However, sex differences in gene expression profiles in the brains of vertebrates are only beginning to be understood. Fishes provide a unique complement to studies of sex differences in mammals and birds given that fish show extreme plasticity and lability of sexually dimorphic characters and behaviors during development and even adulthood. Hence, teleost models can give additional insight into sexual differentiation. The goal of this study is to identify neurotranscriptomic mechanisms for sex differences in the brain.

**Results:**

In this study we examined whole-brain sex-biased gene expression through RNA-sequencing across four strains of zebrafish. We subsequently conducted systems level analyses by examining gene network dynamics between the sexes using weighted gene coexpression network analysis. Surprisingly, only 61 genes (approximately 0.4% of genes analyzed) showed a significant sex effect across all four strains, and 48 of these differences were male-biased. Several of these genes are associated with steroid hormone biosynthesis. Despite sex differences in a display of stress-related behaviors, basal transcript levels did not predict the intensity of the behavioral display. WGCNA revealed only one module that was significantly associated with sex. Intriguingly, comparing intermodule dynamics between the sexes revealed only moderate preservation. Further we identify sex-specific gene modules.

**Conclusions:**

Despite differences in morphology, physiology, and behavior, there is limited sex-biased neural gene expression in zebrafish. Further, genes found to be sex-biased are associated with hormone biosynthesis, suggesting that sex steroid hormones may be key contributors to sexual behavioral plasticity seen in teleosts. A possible mechanism is through regulating specific brain gene networks.

**Electronic supplementary material:**

The online version of this article (doi:10.1186/1471-2164-15-905) contains supplementary material, which is available to authorized users.

## Background

Males and females differ in a number of characteristics ranging from morphology to behavior to physiology. Some traits are almost exclusively observed in one sex (e.g. genitalia). Other traits show sex bias in which they are displayed by both males and females but on average show higher expression in one sex (e.g. some behaviors, context-dependent hormone and gene regulation). Regardless of the degree of bias, understanding the origin and maintenance of sex differences has important evolutionary and biomedical consequences [1–4].

The brain represents a key site of integration for environment and experiential information, resulting in changes in physiology and behavior. In mammals and birds, sex differences in the brain are mostly due to the organizational and activational effects of sex steroid hormones and hormone-independent genetic mechanisms of sex chromosomes [1, 3, 5, 6]. While mammals and well-studied species from other taxa show relatively conserved sex determination patterns characterized by gonochorism, teleost fishes exhibit a high degree of sexual plasticity [7]. Teleost fishes display temperature-dependent, heterogenic, polygenic, and socially-controlled sex determination systems [7, 8]. Even in teleost species that exhibit genotypic sex determination, sex ratios can still be heavily skewed with hormone exposure before sexual maturation [9, 10]. The most dramatic example of plasticity is seen in several families of fishes where mature adults can undergo functional sex change in response to changes in their social environment [7]. Hence, teleost fishes represent unique models that can give insight into sexual lability and sex differences in the brain.

Although there are sexual dimorphisms in zebrafish behaviors and morphology [11, 12], the genetic and hormonal bases are not well understood. Zebrafish do not exhibit strong sex determining gene cascades (e.g. *sry* in mammals) or sexually dimorphic chromosomes [13–15]. Recently, it was documented that zebrafish possess a polygenic sex determination system and sex-associated chromosomal regions are not fixed for the species [13, 15–17]. While zebrafish have been developed as a model system for developmental, toxicological and biomedical studies [17–24], few studies have examined sex differences in this species.

As the genomes between the sexes are largely similar, observed sexual dimorphisms can arise and be maintained through differences in gene expression [25–27]. A substantial amount of differential regulation occurs across the genome between male and female zebrafish gonads [28, 29]. Differences in gene expression in the brain, gonads, and other tissue can be due to activational effects of hormones. In medaka and other teleost fish, sex steroids will directly alter expression of key genes in the brain in a sex-specific manner that can be both transient and reversible [10, 30, 31]. Studies to date examining genome wide expression differences in the brain have focused on one strain or pooled several strains, possibly resulting in a limited view of sex-biased gene expression [28, 32] (but see [33]). To identify genes that may be important for sex differences associated with the brain (e.g. behavior), we compared basal levels of gene expression in the transcriptomes of both males and females across four strains of zebrafish by RNA-sequencing with the goal of identifying those differences that are consistently present between the sexes. We also assessed differences in gene co-expression networks between the sexes. For two strains (HSB (High Stationary Behavior), LSB (Low Stationary Behavior)) with documented sexual dimorphism in stress-related behaviors [11], we assessed whether the expression levels of select genes are associated with individual variation in behavior in each sex.

## Results and Discussion

### Whole-brain transcriptome patterns show little sex bias

**Figure 1:**
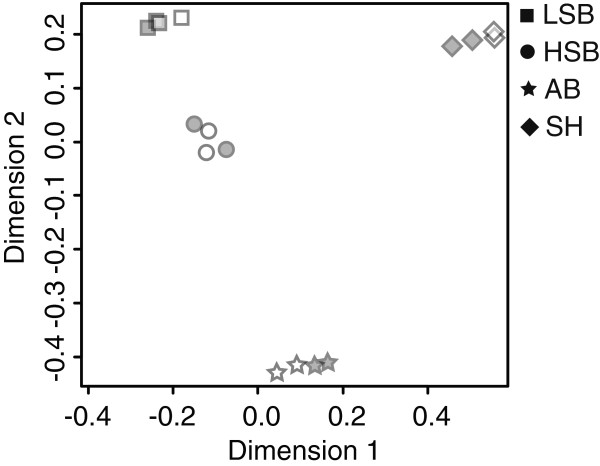
**Multidimensional scaling plot of all genes for each sample.** Square, circle, star, and diamond represents the LSB, HSB, AB, and SH strains, respectively. Male and female samples are represented by open and filled symbols, respectively.

**Figure 2:**
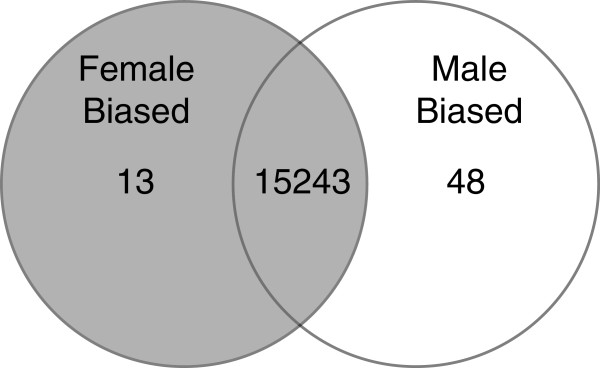
**Venn diagram of sex-biased genes.** After controlling for strain differences, less than 0.5% of transcriptome was differentially expressed between the sexes. See Additional file 1 for gene identities.

In this study we used RNA-sequencing and subsequent bioinformatic analyses to compare the neurotranscriptomes of four strains of zebrafish (AB, SH, HSB, LSB) to identify sex-biased gene expression patterns. Multidimensional scaling analysis revealed that the samples clearly clustered together by strain rather than sex (Figure 1). Of the 15,304 protein coding genes analyzed, 61 showed significant differences between the sexes after controlling for strain differences (Figure 2, Additional file 1). The zebrafish brain shows a substantially lower number of sex-biased genes compared to the gonads or liver [28, 29, 34, 35] and the number of differentially expressed genes in the brain in this study is consistent with other studies [28, 32, 33]. We speculate that having the majority of the genes displaying similar basal level expression between males and females may be an important factor for sexual lability in fishes. If the male and female zebrafish brain is largely similar at the basal transcript level, behavioral and physiological sex differences may be more easily facilitated by other factors such as the hormonal, ecological, or social environment.

Of note, we observed significant sex-biased expression in genes associated with sex steroid production (*cyp19a1b*, *hsd17b3*) and reproduction (*igf1*, *ptgdsb*) across strains (Additional file 1). Brain aromatase (*cyp19a1b*) was female-biased whereas the enzyme that converts androstenedione to testosterone, 17-beta hydroxysteroid dehydrogenase 3 (*hsd17b3*), was male-biased. Brain aromatase and 17-beta hydroxysteroid dehydrogenases have been implicated in a variety of processes ranging from modulating sexual behavior to neural plasticity in teleosts and other species [10, 31, 36, 37]. Sex-biased expression of genes encoding proteins that aromatize androgens (*cyp19a1b*) or aid in synthesizing testosterone (*hsd17b3*) suggest localized neurosteroid production likely contributes to sex differences and lability. Additionally, both insulin-like growth factor 1 (*igf1*) and prostaglandin D2 synthase (*ptgdsb*) in the brain are modulated by sex steroids, alter neural plasticity, and are linked to sexual behavior [38–40]. It is possible that these genes may be important in maintaining sex differences in reproductive behavior in zebrafish.

An enzyme that activates thyroid hormone by converting thyroxine (T4) to triiodothyronine (T4), deiodinase 2 (*dio2*), showed male-biased expression (Additional file 1). Localized thyroid hormone action is critical for normal brain development (reviewed in [41]). Given teleost brains show continuous neurogenesis even as adults [42, 43], we hypothesize that dio2 may help promote sex-specific neural circuits and behavioral plasticity. Thyroid hormone has been shown to alter neural plasticity in a sex-specific manner in rats [44]. Surprisingly, *dio2* is the only gene that was similarly differentially expressed across two of the three other studies of genomic analyses of sex differences in the zebrafish brain. This suggests that sex-biased dio2 activity is conserved in zebrafish. Of note, the other genomic studies used different lines than those used here or did not distinguish lines of zebrafish in their analyses [28, 32, 33]. Since we have demonstrated that there are substantial differences in gene expression by line (Figure 1), it is possible the minimal overlap across studies is due to line differences. Future studies should account for potential line effects in their analyses and interpretations.

Gene ontology analyses of all differentially expressed genes show that these are generally associated with the extracellular matrix, collagen, and isoprenoid and retinoid binding (Table 1). When taking into account the direction of expression, approximately 75% (48/61) of the differentially expressed genes showed male-biased expression (Additional file 1). The explanation for the majority of differentially expressed genes being male-biased is unclear. In mammals, birds and other species with genotypic sex determination systems, potential causes for this bias could be dosage compensation or sex chromosome effects [3, 25, 45, 46]. For zebrafish, these are unlikely as no heterogametic chromosomes have been identified and they possess a polygenic sex determination system [13, 15, 16]. Further, the location of the sex-biased genes in this study is not clustered into the identified sex-associated regions in zebrafish [13, 16] (Additional file 2). The 48 male-biased genes show an over-enrichment of extracellular matrix part and collagen gene ontology terms. The 13 female-biased genes represent over-enriched gene ontology terms comprised of isoprenoid and retinoid binding. We acknowledge the challenging nature of interpreting sex-biased gene ontology terms. Nonetheless, these gene ontology terms can be broadly associated with synaptic plasticity [47–49] and may be involved in maintaining the behavioral differences observed between sexes.

**Table 1:** **Significantly overrepresented gene ontology terms for genes showing sex-biased expression across all four zebrafish strains**

Category	Gene ontology term	ID	FDR corrected p-value
CC	extracellular matrix part	GO:0044420	1.82E-03
CC	collagen	GO:0005581	8.96E-03
CC	extracellular region	GO:0005576	4.88E-04
MF	structural molecule activity	GO:0005198	4.69E-02
MF	extracellular matrix structural constituent	GO:0005201	2.95E-02
MF	isoprenoid binding	GO:0019840	1.51E-03
MF	retinoid binding	GO:0005501	1.51E-03

*CC* Cellular Component, *MF* Molecular Function.

**Figure 3:**
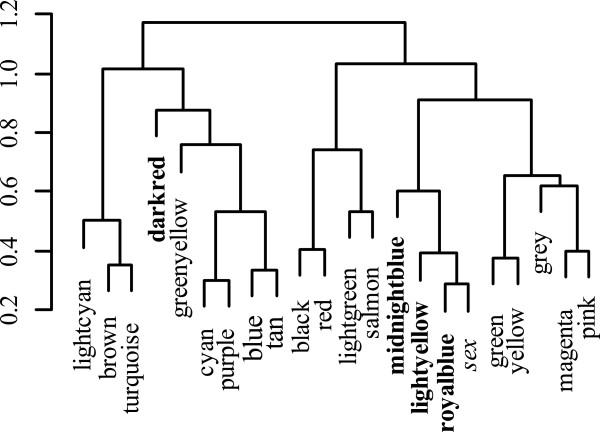
**Hierarchical eigengene diagram of all samples.**All modules were obtained from WGCNA analysis. Modules in bold show a significant association with sex (p < 0.05).

Weighted gene coexpression network analysis (WGCNA) showed that the zebrafish brain transcriptome can be clustered into 21 modules (Figure 3, Additional file 3). Of these modules, the light yellow (p = 0.01), royal blue (p = 0.003), midnight blue (p = 0.005), and dark red (p = 0.03) modules were significantly associated with sex. However, only the midnight blue module shows a strong trend after a Benjimini-Hochberg correction (p_FDR light yellow_ = 0.07, p_FDR royal blue_ = 0.063, p_FDR midnight blue_ = 0.052, p_FDR dark red_ = 0.16). The midnight blue module consists of 72 genes but gene ontology analysis reveals no significantly over-enriched terms. Within the midnight blue module there is a significant and positive correlation between gene significance for sex and module membership (r = 0.31, p = 0.008). This suggests that genes more central to the network (i.e. highly connected) are also strongly associated with sex differences. Of note, *dio2* and *igf1* are among the top 10 most connected genes in the midnight blue module (top 10 genes in decreasing module membership: *slc25a18*, *si:ch211-131 k2.2*, *ckmt1*, *olig2*, *dio2*, *igf1*, *inhbaa*, *gdpd5a*, *nkx6.2*). Across all four modules, 26 genes also showed a significant sex-bias through differential gene expression analysis (Additional file 3). These 26 genes had a significantly higher module membership relative to other genes in the four modules (t = 6.439, p = 1.01 * 10^−9^). This indicates that these sex-biased genes are highly connected within the module, which suggests they are more central to the network (e.g. “hub” genes) and have the potential to be key regulators of the gene co-expression network. The identification of these 26 genes (including genes involved with hormone biosynthesis) as being associated with sex differences in our two independent analyses suggest they may be more important in basal level sex differences and consistent with the idea of neurosteroid production contributing to sexual plasticity in teleost fishes.

### No correlation between individual variation in basal level gene expression and behavior

**Figure 4:**
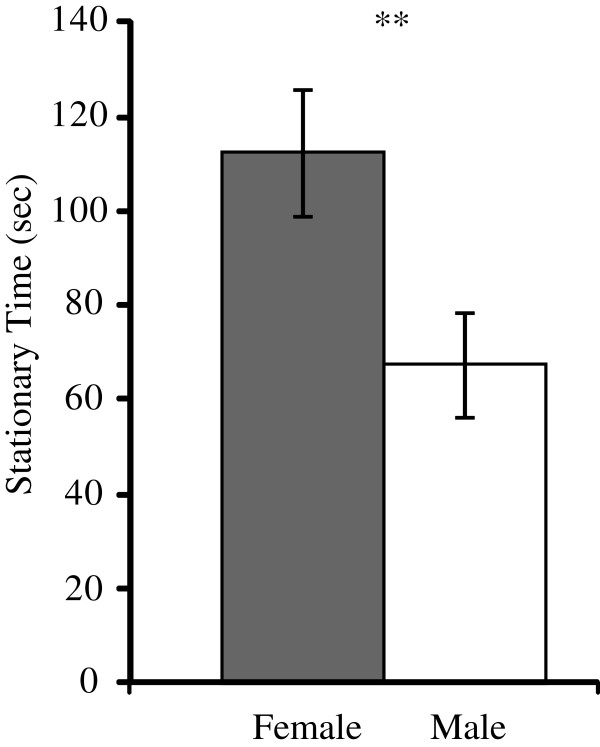
**Time spent stationary in females and males.** Gray and white bars are females (n = 54) and males (n = 58), respectively. Error bars represent standard error. **, p < 0.01.

**Figure 5:**
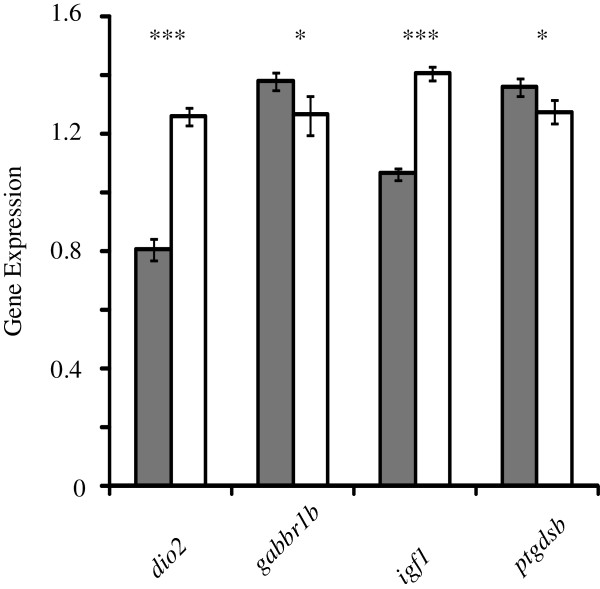
**Significantly differentially expressed genes by qRT-PCR.** Gray and white bars represent female (n = 18) and males (n = 18), respectively. Gene expression was normalized to a housekeeping gene. Error bars represent standard error. ***, p < 0.001; *, p = 0.05.

Females spent significantly more time stationary than males in the open field test (F = 8.413, p = 0.005, Figure 4) after controlling for strain differences. There was no significant strain x sex interaction effect (F = 0.595, p = 0.442) indicating that females spent more time stationary than males in both the LSB and HSB strain. This is consistent with our previous study on earlier generations of the lines [11]. However, there was no significant correlation between expression of any of the genes analyzed by qRT-PCR (*cyp19a1b*, *cfos*, *dio2*, *igf1, gabbr1a*, *gabbr1b*, *ptgdsb*, and *pmchl*) and stationary time in either sex (Additional file 4). The gene *dio2* (F = 81.686, p = 1.2 * 10^−10^) and *igf1* (F = 147.582, p = 1.2 * 10^−13^) showed male-biased expression whereas *ptgdsb* (F =2.837, p = 0.05) and *gabbr1b* (F = 2.862, p = 0.05) showed female-biased expression (Figure 5). The other genes, *cfos* (F = 0.504, p = 0.242), *cyp19a1b* (F = 0.489, p = 0.245), *gabbr1a* (F = 0.341, p = 0.282), and *pmchl* (F = 0.68, p = 0.208) did not show sex-biased expressions. Overall, the expression (log_2_(female expression/male expression)) of all eight genes is consistent between qRT-PCR and RNA-sequencing (r = 0.847, p = 0.008). These results suggest that within-sex variation in the degree of stress and anxiety-related behavioral displays in our HSB and LSB lines is not linearly related to basal levels of the measured genes. It is possible that our measured sex-biased genes (*dio2*, *igf1*, *ptgdsb*, *gabbr1b*) have a threshold effect that facilitates behavioral sex differences. However, we cannot rule out a potential linear relationship for other genes. A multidimensional study identifying markers for anxiety in male rodents suggested cfos and gabbr1 receptor as top candidates [50]. Surprisingly, the expression of these genes was not correlated with our anxiety-related behavioral measure in either sex. Species differences or an unidentified non-linear relationship may account for this observation.

### Gene coexpression network interactions differ between the sexes

WGCNA analyses revealed that the female and male zebrafish brain transcriptomes can be clustered into 25 and 35 modules, respectively (Additional file 5, Additional file 3). In females, 12 of the 25 identified modules showed strong preservation in males. Three modules, however, showed very weak preservation (i.e. unique to females) in males (Additional file 5: Figure S1A). These modules consisted of 418 genes and gene ontology analysis showed no terms were over-enriched. In males, 13 of the 35 modules identified were strongly preserved in females but seven modules showed very weak preservation (i.e. unique to males) (Additional file 5). The seven modules represent 311 genes but do not show over-enrichment of any gene ontology terms. Although the majority of the genes are expressed at a similar level (Additional file 1), network analyses suggest that the genes are largely co-regulated in different ways (Additional file 5) in males and females. We hypothesize that the modules weakly preserved in the opposite sex, when subjected to hormonal, ecological, or social environmental variation, may facilitate the flexibility of sex-specific behavior and physiology in teleosts.

**Figure 6:**
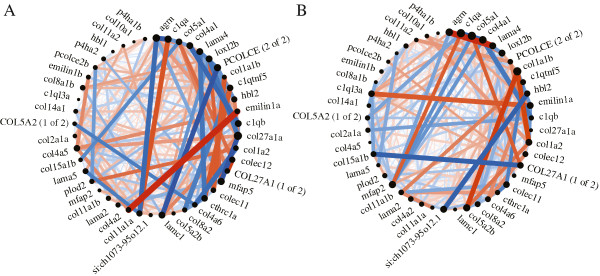
**Extracellular matrix gene coexpression network.** Genes associated with extracellular matrix showed moderate preservation in direction of correlation (color, red = r > 0, blue = r < 0)), correlation coefficient (thickness = | r |), and network centrality (diameter of black circle) between the **(A)** females and **(B)** males.

To compare network properties between categories of significantly over-enriched gene ontology terms (Table 1), we assessed the preservation of gene-expression network interactions between the sexes. Genes associated with the extracellular matrix showed moderate preservation between males and females (Zsummary score = 5.97, Figure 6). Not only do genes associated with the extracellular matrix show sex-biased expression, but the coexpression network also differs between the sexes. The differences in presumed co-regulation of these genes may explain the sex-biased expression. The extracellular region (Zsummary score = 14.44) and structural molecule activity (Zsummary score = 17.00) gene ontology terms displayed very high preservation of gene expression interactions between the sexes (Additional files 6 and 7). Despite sex-biased expression in genes associated with the extracellular region and structural molecule activity, the gene coexpression networks are largely similar between the sexes. The mechanism warrants further study.

## Conclusions

Sex differences in morphology, physiology and behavior are prevalent across many species. In teleost fishes, sexual plasticity is often very high. As an initial attempt to understand a mechanism of sexual plasticity in fish, we characterize sex differences in basal gene expression levels, gene coexpression networks, and stress and anxiety-related behavioral responses across several lines of zebrafish. We identified that a small fraction (0.4%) of the neural transcriptome is differentially expressed at the basal level after controlling for line differences. Interestingly, observing less sexual dimorphism in gene expression in the brain relative to other tissues is consistent with studies in a wide variety of taxa from fruit flies to birds and to rodents [4, 26, 45, 46, 51]. Sex-biased genes in zebrafish are associated with steroid hormone biosynthesis and synaptic plasticity suggesting local neurosteroid production to be a key modulator of the sexual plasticity observed in adult teleosts. Since we did not detect any overrepresentations of general biological, cellular, or molecular pathways in the sex-specific modules, with approximately half the modules showing moderate to weak preservation across the opposite sex, it is suggestive that certain genes in the transcriptome are being co-regulated in a sex-specific manner. Of the genes analyzed, we did not observe any correlation between basal level of expression and stationary behavior. The presence of only modest differences in gene expression across the brain transcriptome coupled with sex-specific gene coexpression networks possibly allows for sexual plasticity in teleosts to be easily modulated by hormonal, ecological, or social factors.

## Methods

### RNA sequencing analysis

In four lines of zebrafish we quantified and compared the whole-brain transciptomes in males and females. All fish were maintained in mixed sex 100-liter tanks on a recirculating filtration system at 28°C with a 12:12 light dark cycle and fed daily. Two zebrafish lines, AB and Scientific Hatcheries (SH) were purchased from commercial suppliers, Zebrafish International Resource Center and Scientific Hatcheries, respectively. The other two lines (HSB, LSB) originated from wild caught individuals and were produced through selective breeding (as described in [11]). The SH and AB lines were maintained in our laboratory for one and four generations, respectively. The HSB and LSB individuals were six generations removed from the wild. All individuals (n = 20 for each sex for each line) were 17 weeks post-fertilization and sexually mature. Sex was assigned by confirming presence of testis or ovaries on dissection. Fish were removed from their home tanks and quickly sacrificed between 09:00 – 12:00. Brains were removed in under three minutes following removal from the tank, stored in RNAlater (Ambion, Austin TX) at 4°C overnight and then stored at −80°C until RNA extraction. Due to limited numbers of fish in the HSB and LSB lines, 15 of the individuals we sampled from each of these lines (of 40 total) had undergone behavioral testing three weeks prior (see below). All procedures and protocols in this study were approved by the North Carolina State University Institutional Animal Care and Use Committee.

RNA extraction and RNA-sequencing followed our previously established protocol [52]. Briefly, RNA was extracted from 160 individuals (20 individuals of each sex for each strain) using RNeasy Plus Mini Kit (Qiagen). As the goal of this part of the experiment was to assess a general effect of sex on the transcriptomes, for each strain we pooled one microgram of total RNA from 10 same sex individuals into one biological replicate. This resulted in eight biological replicates for each sex (two biological replicates for each strain). RNA quality was assessed with an Agilent 2100 Bioanalyzer (Agilent) and all samples had RNA integrity numbers (RIN) above 8.0. We followed the manufacturer’s protocol for cDNA library preparation (TruSeq RNA Sample Prep V2, Illumina) and submitted our samples to the Genomic Sciences Laboratory at North Carolina State University for 72 bp single-end RNA sequencing (Illumina GAIIx). Utilizing a balanced block design [53], all samples were multiplexed and run across 16 lanes. We combined reads across all lanes that passed default quality control filters, which resulted in approximately 52 million reads per biological replicate (ranging from 34–65 million reads). This data is accessible through NCBI’s Gene Expression Omnibus (GSE61108). We aligned the reads to the *Danio rerio* genome (assembly Zv9 [17], release 71) using GSNAP [54] with default parameters. We used HTSeq to quantify the number of reads aligned to each gene using the “union” mode. We employed a two-factor design using EdgeR [55] to assess differential expression of protein-coding genes between the sexes with strain as a cofactor. We used gProfiler [56, 57] to determine significantly over-enriched gene ontology (GO) terms. We utilized the default false discovery rate (FDR) corrections in both EdgeR and gProfiler. Statistical significance was defined as p_FDR-corrected_ < 0.05.

### Gene coexpression network analysis

To characterize the gene expression network dynamics we utilized weighted gene co-expression network analysis (WGCNA [58]) using normalized expression counts from all the genes that underwent differential expression analysis in edgeR. WGCNA clusters together highly correlated genes into modules, which can then be used to assess a variety of attributes (see [58] and references within for full details). We assessed network dynamics with two goals in mind: 1) identify modules associated with sex and 2) identify modules unique to one sex (i.e. not preserved across sexes). WGCNA analysis revealed that one of the LSB strain female biological replicates was an outlier and we removed that sample from all WGCNA analyses. To identify modules associated with sex, we ran WGCNA on all 15 samples. Subsequently within modules that passed FDR correction, we assessed the relationship between gene significance for sex and module membership. Module membership represents the correlation of the module eigengene and the gene expression profile and is used as a proxy for measuring how central the gene is within the module (see [58] for more details). We ran separate WGCNA analyses for each sex (n = 7 for females and n = 8 for males) to assess module preservation across sex. In all cases we adjusted soft-threshold (β) values to ensure an approximate scale-free topology [59], set the minimum module size to 30 and a dynamic tree cut height to 0.3 to ensure a larger number of genes in each module to assess intramodule dynamics, and used the default parameters for all other WGCNA settings. Statistical significance of modules associated with sex was determined when p < 0.05 using a Benjamini-Hochberg correction. Module preservation statistics across sex were conducted and defined as in [60]: Preservation Z-Summary scores greater than 10, between 10 and 2, and less than 2 are designated as strongly, moderately, and weakly (i.e. unique) preserved. Preservation Z-Summary is a composite summary statistic that includes measures of density and connectivity between networks and is used to measure the preservation of network properties within a module or set of genes between two networks (see [60] for more details).

We also assessed the preservation of genes assigned to the gene ontology terms extracellular matrix part (GO ID: 0044420), extracellular region (GO ID: 0005576), and structural molecule activity (GO ID: 0005198) between males and females. We selected these gene ontology terms because they were significantly over-enriched from our edgeR analysis (see Results) and were parent terms to the other over-enriched terms. Although isoprenoid binding (GO ID: 0019840) is a parent term, we did not analyze preservation between sexes because this GO term comprises only three genes in zebrafish. Analysis and visualization of the preservation of these genes between the sexes followed an established protocol [60]. We defined preservation across sexes as above.

### Behavioral analysis

We exposed males and females from each of the HSB and LSB lines (n = 54 for females, n = 58 for males) to an open field test using established methods [11, 61]. Briefly, we exposed individual fish to a 30 × 30 × 10 cm (width × length × height) arena filled with 4 liters of aquarium system water (water used to house fish). During the five minute trial we recorded the amount of time spent stationary (moving less than 0.1 cm/s) using automated software (TopScan Lite, Reston, VA, USA). Of these fish, 18 of each sex were from the same cohort as those used in the RNA-sequencing analyses. Nine fish of each sex from each line were individuals seven generations removed from the wild and sacrificed immediately after the behavioral assay and prepared for quantitative reverse transcriptase PCR analysis (see below). We chose to examine only HSB and LSB lines because we have previously shown that females show higher stress and anxiety-related behavioral displays than males in these lines [11]. We assessed differences in stationary time using a general linear model with sex and strain as cofactors (SPSS version 20).

### Quantitative reverse-transcriptase PCR

For 36 fish (18 of each sex) we measured the expression of *cyp19a1b*, *cfos*, *dio2*, *igf1*, *gabbr1a*, *gabbr1b*, *ptgdsb*, and *pmchl* through quantitative reverse transcriptase PCR (qRT-PCR). We selected these genes because they show sex differences in zebrafish from our RNA-sequencing results (*cyp19a1b*, *dio2*, *igf1, ptgdsb*, *pmchl*) or are associated with stress and anxiety related behaviors in other species [50]. All fish were immediately sacrificed after open field testing (see above). Preparation, execution, and anlaysis of the qRT-PCR followed methods described previously [52]. Briefly we homogenized tissue in Trizol (Invitrogen) and extracted the RNA through column filtration (RNeasy Plus Mini Kit, Qiagen). RNA was subsequently converted to cDNA (SuperScript III First-Strand Synthesis System for qRT-PCR, Invitrogen) and purified (Amicon Ultra −0.5 mL 30 K Centrifugal Filters, Millipore). We ran qRT-PCR reactions on an ABI 7900HT Fast Real-Time PCR system (Applied Biosystems) using SYBR Select (Applied Biosystems). Primers either spanned exon-exon junctions or the amplicon spanned two exons with an included intron region over 1 kilobase. Each sample was run in triplicate (see Additional file 8 for primer sequences, amplicon lengths, and qRT-PCR reaction parameters). Gene expression was normalized to the expression of a housekeeping gene (*ef1a*). Transcript abundances for *ef1a* have been shown to be stable across sex and age in zebrafish [62]. To assess differences in gene expression between the sexes we used a general linear model with strain as a cofactor. We predicted that qRT-PCR patterns would follow those seen in the RNA-sequencing analysis and assess statistical significance using one-tailed p-values. We used Pearson’s correlations to assess relationships between gene expression and stationary behavior and determined significance with two-tailed p-values. Statistical analyses were performed in SPSS (version 20).

## Availability of supporting data

The data set(s) supporting the results of this article is(are) included within the article (and its additional file(s)). Data is also accessible through NCBI’s Gene Expression Omnibus (GSE61108).

## Electronic supplementary material

Additional file 1: **Quantification (counts per million) and statistical results of all genes that underwent differential expression analysis.** Male-biased and female-biased genes are highlighted in the Sex-biased Genes worksheet in blue and red, respectively. (XLSX 5 MB)

Additional file 2: **Labeling of sexually dimorphic genes on zebrafish chromosomes.** Genomic location of the differentially expressed genes (red lines) does not strongly correspond to putative sex-associated regions (gray [13]). (PDF 100 KB)

Additional file 3: **Module classifications of the zebrafish (both male and female), male only, and female only transcriptomes.** The 26 genes in common across the four modules associated with sex differences and genes showing sex-biased expression are highlighted in yellow within the male and female combined classification worksheet. Each classification is on a separate worksheet. (XLSX 1 MB)

Additional file 4: **Correlation between stationary time and gene expression measured by qRT-PCR for both females and males.** (DOCX 15 KB)

Additional file 5: **Analysis of module preservations across each sex.** Preservation scores for the 25 and 35 modules identified in the A) female and B) male transcriptomes, respectively. Preservation score designations follows that in [60]. (PDF 124 KB)

Additional file 6: **Extracellular region gene coexpression networks.** Genes associated with extracellular region and structural molecule activity showed high preservation in direction of correlation (color, red = r > 0, blue = r < 0)), correlation coefficient (thickness = | r |), and network centrality (diameter of black circle) between females and males. (PDF 2 MB)

Additional file 7: **Structural molecule activity gene coexpression networks.** Genes associated with extracellular region and structural molecule activity showed high preservation in direction of correlation (color, red = r > 0, blue = r < 0)), correlation coefficient (thickness = | r |), and network centrality (diameter of black circle) between the females and males. (PDF 1 MB)

Additional file 8: **qRT-PCR primer characteristics.** (DOCX 16 KB)
