# Supplementary figures and images for: Limited sex-biased neural gene expression patterns across strains in Zebrafish (Danio rerio)
Source: BMC Genomics. 2014 Oct 17;15(1):905. doi: 10.1186/1471-2164-15-905 (PMC4216363; doi:10.1186/1471-2164-15-905)

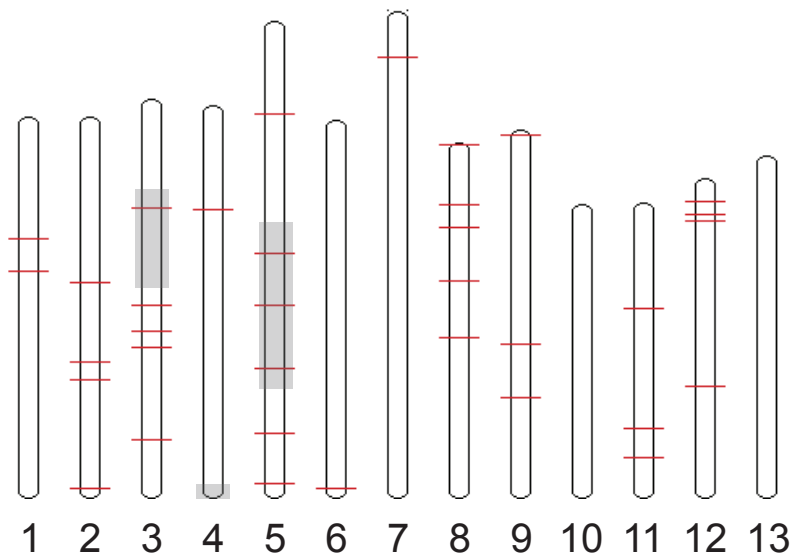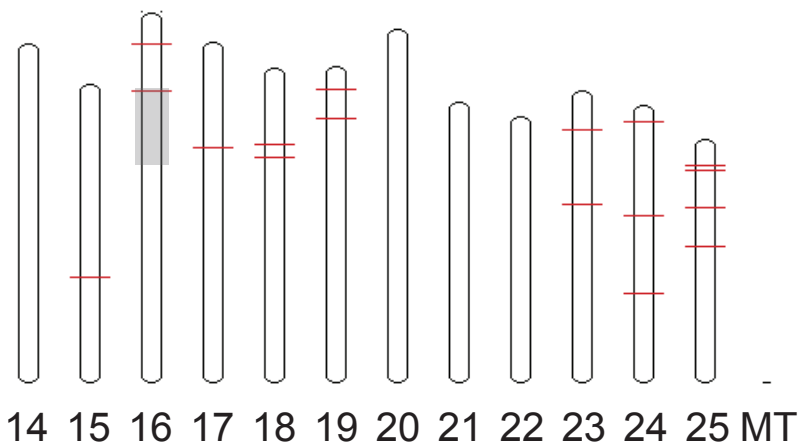

Supplement: Supplementary file 2 — Additional file 2: Labeling of sexually dimorphic genes on zebrafish chromosomes. Genomic location of the differentially expressed genes (red lines) does not strongly correspond to putative sex-associated regions (gray [13]). (PDF 100 KB) [file 12864_2014_6615_MOESM2_ESM.pdf]

a)

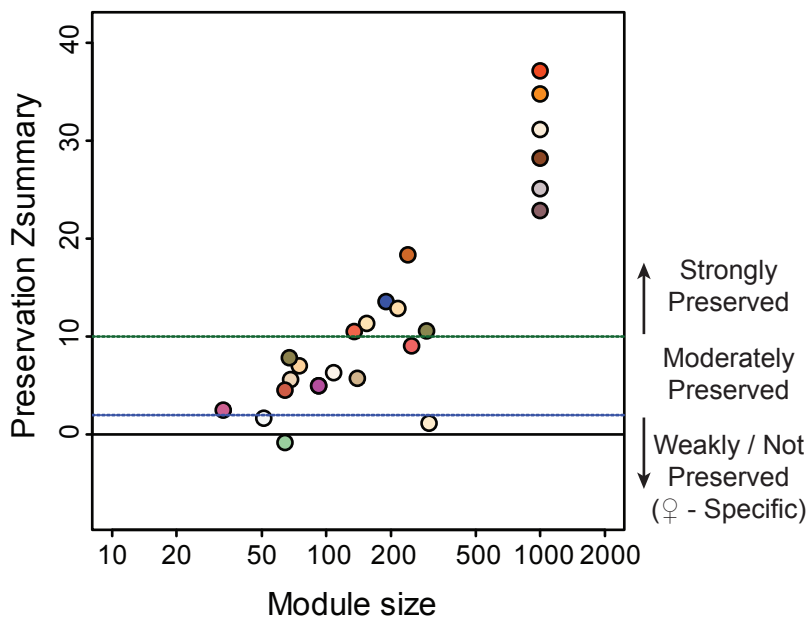

b)

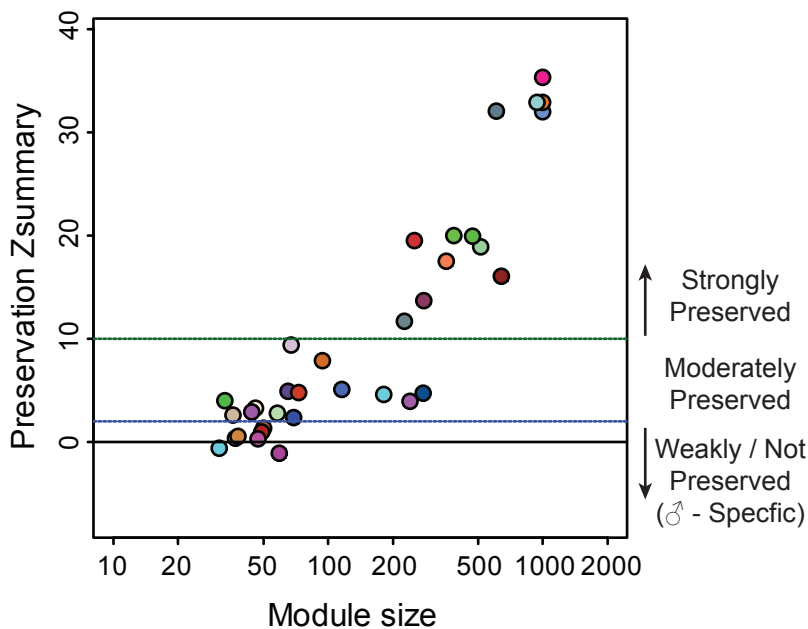

Supplement: Supplementary file 5 — Additional file 5: Analysis of module preservations across each sex. Preservation scores for the 25 and 35 modules identified in the A) female and B) male transcriptomes, respectively. Preservation score designations follows that in [60]. (PDF 124 KB) [file 12864_2014_6615_MOESM5_ESM.pdf]

Female

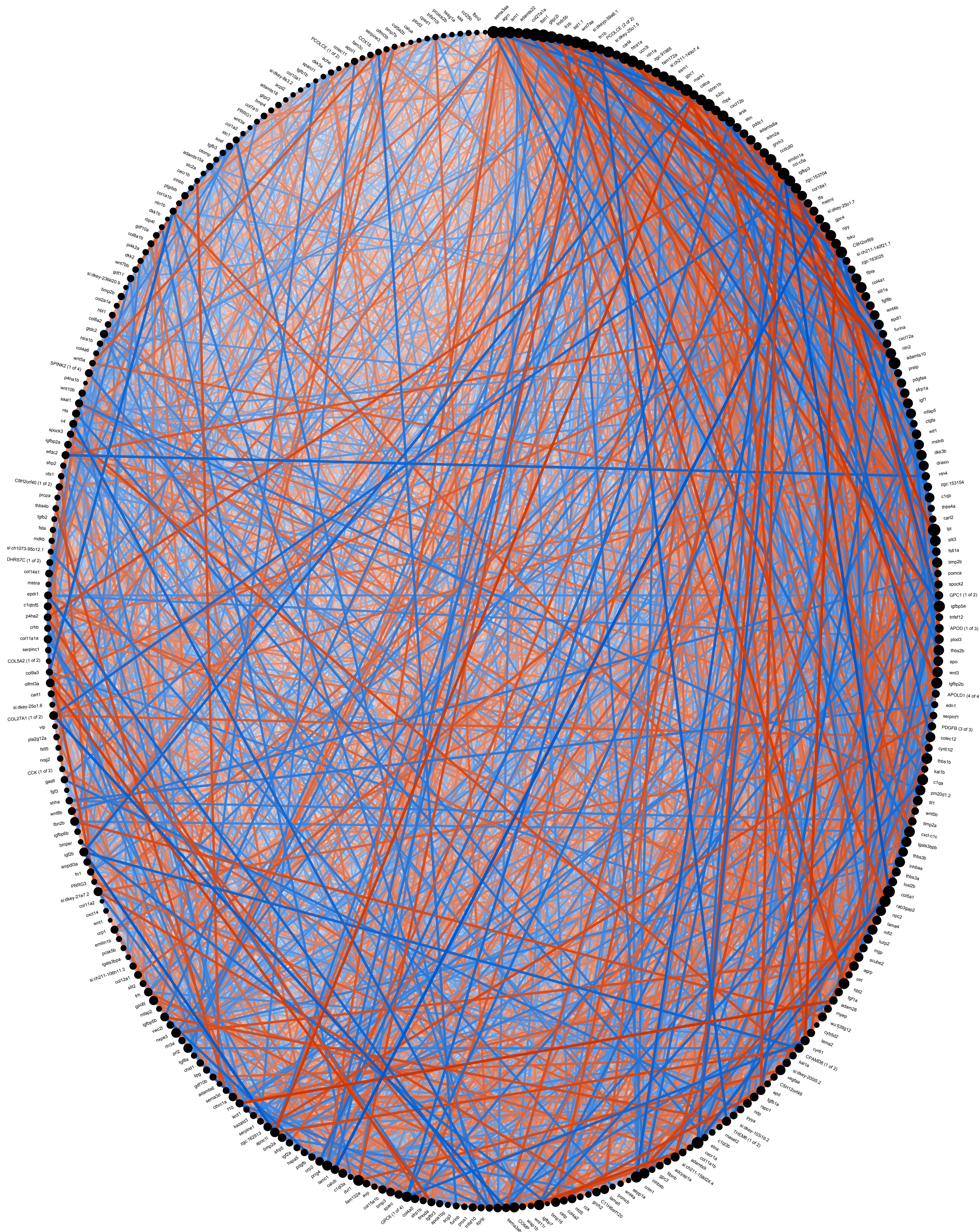

Male

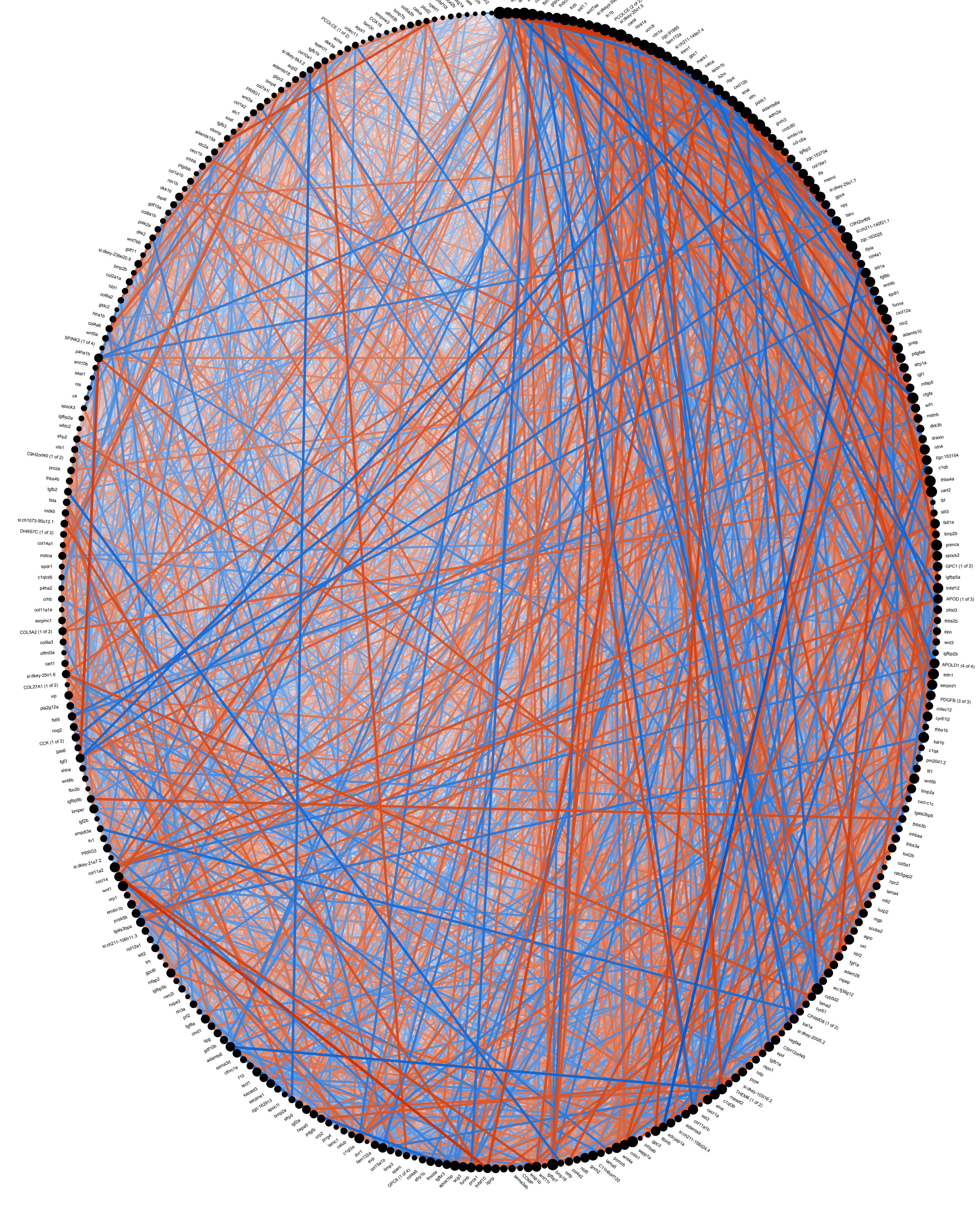

Supplement: Supplementary file 6 — Additional file 6: Extracellular region gene coexpression networks. Genes associated with extracellular region and structural molecule activity showed high preservation in direction of correlation (color, red = r > 0, blue = r < 0)), correlation coefficient (thickness = | r |), and network centrality (diameter of black circle) between females and males. (PDF 2 MB) [file 12864_2014_6615_MOESM6_ESM.pdf]

Female

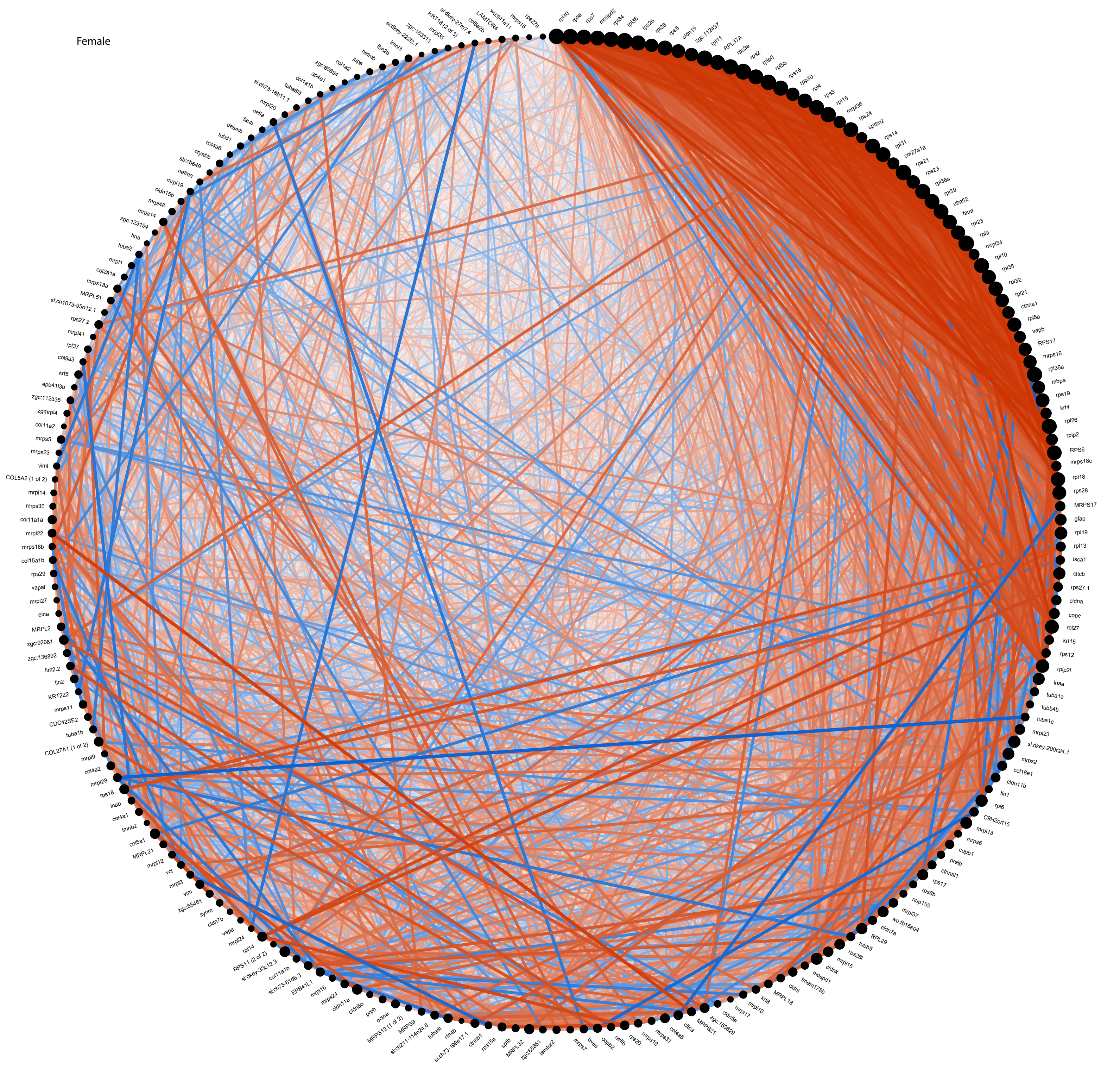

Male

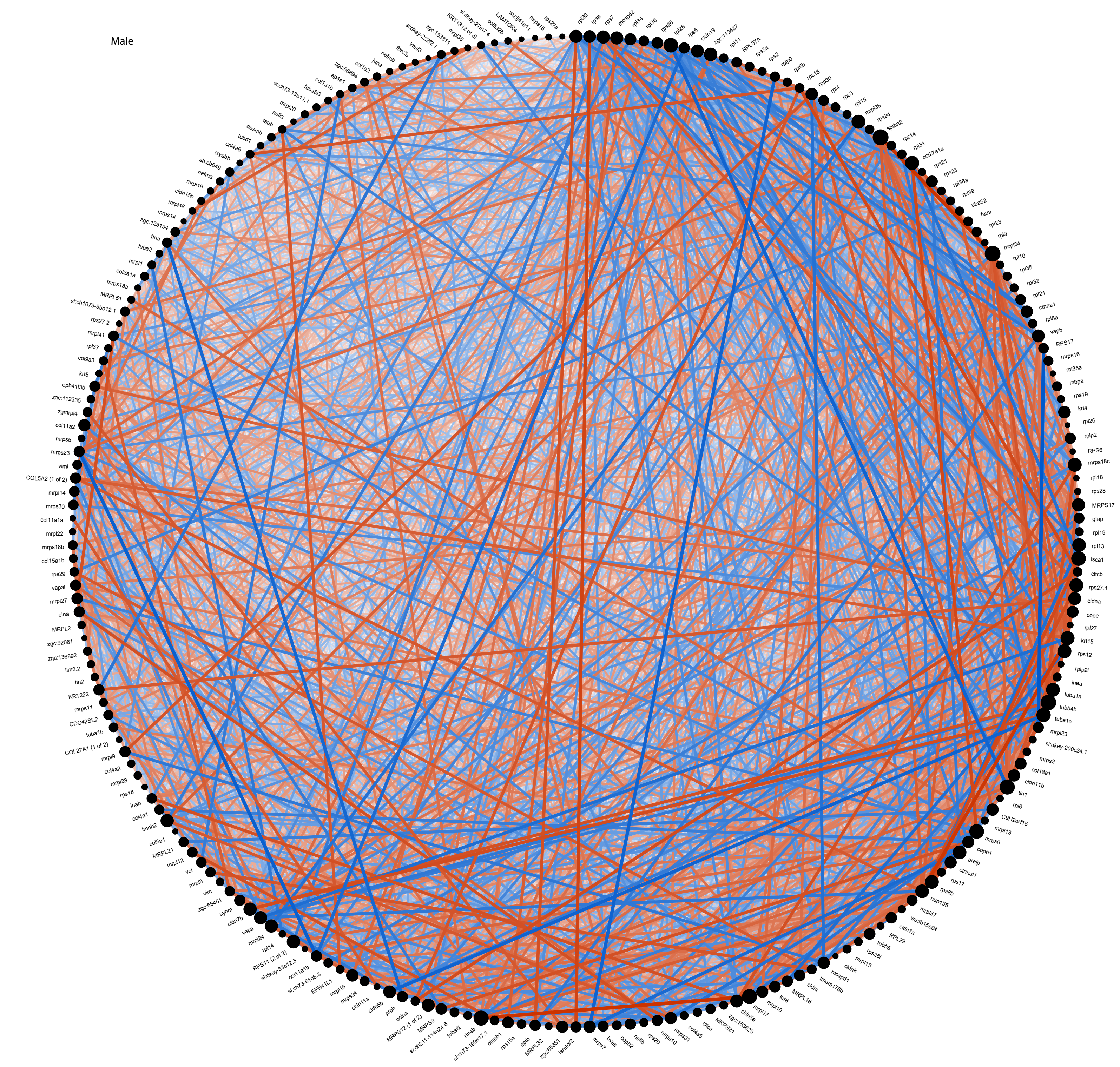

Supplement: Supplementary file 7 — Additional file 7: Structural molecule activity gene coexpression networks. Genes associated with extracellular region and structural molecule activity showed high preservation in direction of correlation (color, red = r > 0, blue = r < 0)), correlation coefficient (thickness = | r |), and network centrality (diameter of black circle) between the females and males. (PDF 1 MB) [file 12864_2014_6615_MOESM7_ESM.pdf]
