# Supplementary material for: Limited sex-biased neural gene expression patterns across strains in Zebrafish (Danio rerio)
Source: BMC Genomics. 2014 Oct 17;15(1):905. doi: 10.1186/1471-2164-15-905 (PMC4216363; doi:10.1186/1471-2164-15-905)
Supplement: Supplementary file 4 — Additional file 4: Correlation between stationary time and gene expression measured by qRT-PCR for both females and males. (DOCX 15 KB) [file 12864_2014_6615_MOESM4_ESM.docx]

Additional file 4: Correlation between stationary time and gene expression measured by qRT-PCR for both females and males.

|  | **Female** | | **Male** | |
| --- | --- | --- | --- | --- |
| **Gene** | **Pearson’s r** | **p-value** | **Pearson’s r** | **p-value** |
| *cyp19a1b* | -0.123 | 0.626 | -0.103 | 0.684 |
| *cfos* | -0.213 | 0.396 | 0.19 | 0.451 |
| *dio2* | 0.266 | 0.286 | -0.079 | 0.755 |
| *igf1* | 0.25 | 0.317 | 0.034 | 0.897 |
| *gabbr1a* | 0.09 | 0.723 | 0.055 | 0.828 |
| *gabbr1b* | -0.151 | 0.549 | 0.288 | 0.247 |
| *ptgdsb* | 0.015 | 0.953 | 0.458 | 0.056 |
| *pmchl* | 0.144 | 0.569 | -0.095 | 0.708 |
