# Supplementary material for: Limited sex-biased neural gene expression patterns across strains in Zebrafish (Danio rerio)
Source: BMC Genomics. 2014 Oct 17;15(1):905. doi: 10.1186/1471-2164-15-905 (PMC4216363; doi:10.1186/1471-2164-15-905)
Supplement: Supplementary file 8 — Additional file 8: qRT-PCR primer characteristics. (DOCX 16 KB) [file 12864_2014_6615_MOESM8_ESM.docx]

Additional file 8: qRT-PCR primer characteristics

| Gene Symbol | Forward Primer | Reverse Primer | Amplicon Length |
| --- | --- | --- | --- |
| CFOS | 5’-TACACTGCAAGCTGAAACTGACCA-3’ | 5’-TTGTGTGCGGCGAGGATGAA-3’ | 114 |
| CYP19A1B | 5’-GTTACAGTCGGTTCCTCTGG-3’ | 5’-GCTCTTCAGGACATGGTACAC-3’ | 146 |
| DIO2 | 5’-CTACAAACAGGTGAAATTGGGCG-3’ | 5’-GGTTGTGGGTCTTACCGCTG-3’ | 101 |
| EF1A | 5’-CCTCTTGGTCGCTTTGC-3’ | 5’-GGTGTGATTGAGGGAAATTCA-3’ | 150 |
| GABBR1A | 5’-CCCAGAGACGGAGGGATACG-3’ | 5’-CGGGCACATCATCAAGCATCT-3’ | 198 |
| GABBR1B | 5’-AGGTGTTGCCGGTGGATTATGAAA-3’ | 5’-TGGAGGATGAACCGTCACACG-3’ | 188 |
| IGF1 | 5’-TGAGATGTGACATTGCCCGC-3’ | 5’-AGCGCATGGTACACTTAAAGACA-3’ | 158 |
| PMCHL | 5’-ACCGCTAAAGCAAACGCTCA-3’ | 5’-CTGGAGCCACCAGGTGTAGA-3’ | 199 |
| PTGDSB | 5’-TGCCTATGACTGACTTCGACCT-3’ | 5’-AGCAGGAACCATCACTCTTTAGG-3’ |  |

qRT-PCR parameters (all genes): 2 min at 50°C, 2 min at 95°C, followed by 40 cycles at 15 sec 95°C and 1 min 60°C. Primer concentration was 5 pmol for EF1A, GABBR1A, IGF1, PMCHL, PTGDSB, and 1.5 pmol for CFOS, CYP19A1B, DIO2, GABBR1B.
